# *Streptococcus milleri* and Recurrent Intra-Abdominal Abscesses: A Case Report and Literature Review

**DOI:** 10.1155/2016/6297953

**Published:** 2016-05-30

**Authors:** Tabitha M. Gana, Olugbenga Awolaran, Sobia Akhtar

**Affiliations:** Department of General Surgery, Doncaster and Bassetlaw NHS Trust, Armthorpe Road, Doncaster DN2 5LT, UK

## Abstract

We report a case of recurrent intra-abdominal abscesses as a postoperative complication following diverticular perforation in which* Streptococcus milleri* (SM) was isolated. SM is evaluated here as a potent pyogenic organism commonly associated with intra-abdominal abscess especially in the postoperative setting. With the commonly adopted conservative management, the challenges of recurrence and prolonged hospital stay experienced in the indexed case as well as many other previous reports are highlighted. We also present a recommendation of the need for a more intensive approach of SM-related abscess drainage along with areas that would benefit further research.

## 1. Introduction

*Streptococcus milleri* (SM) infection (also known as* Streptococcus anginosus*) has been shown to be commonly associated with surgical intervention. These organisms are well known for their very potent pyogenic potential and high abscess recurrence rate despite antibiotic treatment. We report a case of SM isolation from intraabdominal pus following sigmoid diverticular perforation and the challenges involved in the surgical management.

## 2. Case Report

An otherwise fit and healthy 56-year-old man presented to the emergency department with a week history of generalized abdominal pain associated with nausea and anorexia. Examination revealed generalized peritonitis. Erect chest X-ray showed extensive free air under both hemidiaphragms suggesting a perforated viscus while C-reactive protein and white cell count were raised at 368 mg/L and 20.16 × 10^9^/L, respectively.

He was resuscitated with intravenous fluids, analgesia, commenced on empiric antibiotics (1.2 grams of amoxicillin/clavulanic acid and 500 milligrams of metronidazole, three times a day) and scheduled for urgent surgery. Initially, a laparoscopic approach was employed but converted to open due to technical difficulties. Intraoperative findings included multiple intraperitoneal abscess cavities and an inflamed and thickened sigmoid colon. The perforation was not distinctively identified and was thought to be sealed within the thickened sigmoid colon. Hartmann's procedure was performed, abscesses were drained, aspirate was sent for culture, and peritoneum copiously lavaged.

The patient had a slow postoperative recovery on ICU with persistently raised inflammatory markers (C-reactive protein > 200 mg/L and white cell count > 15 × 10^9^/L) which necessitated changing antibiotics to 4.5 grams of piperacillin/tazobactam, three times a day. Peritoneal aspirate was positive for* S. milleri* and a scanty amount of* E. coli* after 48 hours of culture; following this, weight-tailored doses of gentamicin were added. Histopathology analysis of the sigmoid specimen revealed evidence of perforated diverticulum. CT scan on the 5th postoperative day showed extensive multiloculated gas and fluid collection in the left abdomen extending from the diaphragm to the pelvis.

Percutaneous drainage of this collection was performed, following which the patient made remarkable clinical progress initially. However, shortly after, he developed pyrexia and abdominal pain. Repeat CT scan showed a reduction in the size of the left flank and pelvic collections but presence of a left subphrenic collection. Due to lack of clinical improvement over the following days with continued antibiotics, another drain was into the left subphrenic collection. He made good clinical improvement, drain output reduced and inflammatory markers normalized. After a total course of about 2 weeks of IV antibiotics, he had the flank drain removed and was discharged with oral antibiotics. On follow-up in the clinic after 2 weeks, he was clinically well and the subphrenic drain was also removed.

## 3. Discussion

SM organisms are well known for their very potent pyogenic potential and high abscess recurrence rate despite antibiotic treatment. Abscess formation by these organisms has been reported in a wide range of organs and systems; however they cause more intra-abdominal infections compared to other sites [1, 2].

While the exact mechanism of abscess formation by SM organisms is yet to be fully established, this has been linked to their ability to secrete toxins that inhibit polymorphonuclear leukocytes ingestion and also prolong their survival after ingestion [3]. They also produce hydrolytic enzymes such as hyaluronidase which is thought to be responsible for spreading organisms through tissue and assist in the liquefaction of pus [4, 5].

SM infection has been shown to be commonly associated with surgical intervention, especially abdominal procedures. Stezmuller et al. in a study assessing pattern of infection in 24 patients with SM bacteraemia found that up to 15 patients (62.5%) had a surgical procedure before SM-positive blood culture. They noted that SM can be considered a “strong surgical pathogen” and recommended that, with a positive blood culture, a surgical sepsis should be considered. In another report, SM was the commonest organism found in pus culture (73%) following drainage of intra-abdominal abscess complicating appendectomy [6]. Similarly, SM organisms were associated with a sevenfold increase in abscess formation following appendectomy despite antibiotics and consequently increased morbidity and prolonged hospital stay [7]. SM isolation from intra-abdominal pus following sigmoid diverticular perforation is presented here along with the challenges involved in the surgical management. Similar to previous reports, extensive postoperative multiloculated intra-abdominal abscesses were found which necessitated repeated percutaneous drainage and prolonged antibiotic use.

In terms of antimicrobial therapy, SM organisms are largely susceptible to penicillins and also show good sensitivity to other beta-lactam antibiotic like cephalosporins and carbapenems [8, 9]. Similar to other reported cases, antibiotics here were tailored to the sensitivity of SM isolated; however the patient did not make significant clinical progress postoperatively until the abscesses were repeatedly drained.

Though it is well established that successful treatment of abscess generally relies on their drainage, evidence points to a need for a closer look into the method of achieving the optimal drainage in intra-abdominal abscess caused by such pyogenic organisms like SM. Current approach of radiologically guided drainage and antibiotics is associated with a high recurrence rate. Ripley et al. in a study of 39 patients with SM pleural space infection retrospectively compared operative drainage (video-assisted thoracoscopic surgery and thoracotomy) with nonoperative drainage (thoracostomy tube and radiologically placed pigtail catheters) [10]. They reported shorter hospital stay and less mortality in the operative compared to the nonoperative group. Their explanation was that SM infections tend to cause loculated collections which are less likely to be amenable to tube or catheter drainage. The intra-abdominal abscess in the case we reported was noted to be similarly multiloculated. It is therefore a plausible extrapolation that an operative, for example, laparoscopic washout may have benefited our patient more; however this requires further studies to validate.

With such robust evidence for SM association with recurrent intra-abdominal abscesses, we suggest that a more intensive approach to drainage may be necessary as conservative use of radiologically placed drains have been met with high recurrence and morbidity. The value of surgical drainage in comparison to radiologic drainage is worth investigation as preliminary evidence has shown that operative drainage offers better outcome in pleural space collections. Other modifications in the management of SM abscess that needs further research into for added benefit include the timing and duration of antibiotics, use of repeated imaging to aid in timely identification of recollection, and optimal size and type of drains to be used.
